# The *agr* Locus Regulates Virulence and Colonization Genes in Clostridium difficile 027

**DOI:** 10.1128/JB.00473-13

**Published:** 2013-08

**Authors:** Melissa J. Martin, Simon Clare, David Goulding, Alexandra Faulds-Pain, Lars Barquist, Hilary P. Browne, Laura Pettit, Gordon Dougan, Trevor D. Lawley, Brendan W. Wren

**Affiliations:** Department of Pathogen Molecular Biology, London School of Hygiene and Tropical Medicine, University of London, London, United Kingdoma; Microbial Pathogenesis Laboratory, Wellcome Trust Sanger Institute, Hinxton, United Kingdomb; Rfam Group, Wellcome Trust Sanger Institute, Hinxton, United Kingdomc; EMBL-European Bioinformatics Institute, Hinxton, United Kingdomd; Bacterial Pathogenesis Laboratory, Wellcome Trust Sanger Institute, Hinxton, United Kingdome

## Abstract

The transcriptional regulator AgrA, a member of the LytTR family of proteins, plays a key role in controlling gene expression in some Gram-positive pathogens, including Staphylococcus aureus and Enterococcus faecalis. AgrA is encoded by the *agrACDB* global regulatory locus, and orthologues are found within the genome of most Clostridium difficile isolates, including the epidemic lineage 027/BI/NAP1. Comparative RNA sequencing of the wild type and otherwise isogenic *agrA* null mutant derivatives of C. difficile R20291 revealed a network of approximately 75 differentially regulated transcripts at late exponential growth phase, including many genes associated with flagellar assembly and function, such as the major structural subunit, FliC. Other differentially regulated genes include several involved in bis-(3′-5′)-cyclic dimeric GMP (c-di-GMP) synthesis and toxin A expression. C. difficile 027 R20291 *agrA* mutant derivatives were poorly flagellated and exhibited reduced levels of colonization and relapses in the murine infection model. Thus, the *agr* locus likely plays a contributory role in the fitness and virulence potential of C. difficile strains in the 027/BI/NAP1 lineage.

## INTRODUCTION

Clostridium difficile is a Gram-positive, anaerobic bacterium that is arguably the most frequent cause of antibiotic-associated colitis and health care-acquired diarrhea worldwide. Disease-causing isolates can produce two exotoxins, TcdA and TcdB, encoded by the 19-kb pathogenicity locus (PaLoc), which interact with the intestinal epithelium and potentially precipitate an acute inflammatory response and even cell death (1–3). Colonization of the intestine by toxigenic C. difficile can be asymptomatic, but following antibiotic treatment, a variety of symptoms, including diarrhea or life-threatening pseudomembranous colitis, can ensue (4). Relapsing disease occurs in up to 20% of patients following termination of treatment with certain first-line antimicrobials, such as vancomycin or metronidazole (5). The global increase in incidence and severity of C. difficile infection over the last decade is linked to the emergence of certain lineages, including the epidemic 027/BI/NAP1 variants (6–11). Transcontinental dissemination of the 027 variant occurred through at least two distinct fluoroquinolone-resistant lineages, with clinical outcomes resulting in longer colonization duration, increased toxin production, and increased relapse and mortality rates (6, 12, 13). This increased disease severity was typified by multiple outbreaks of C. difficile disease between 2003 and 2006, affecting over 300 patients at the Stoke Mandeville hospital, United Kingdom, from which the representative 027 strain, R20291, was isolated (14, 15).

Gene regulatory networks can enable pathogenic bacteria to rapidly adapt to their environment and modulate the expression of virulence-associated factors. So-called two-component systems (TCSs) can play a key role in linking environmental and internal sensing to the control of gene expression. Interestingly, genes with similarity to TCSs, transcriptional regulators, and signaling proteins comprise approximately 10% of C. difficile genomes, yet their contribution to the regulatory mechanisms and virulence within C. difficile are poorly understood (14, 16). The RolA/B TCS of C. difficile has been shown to negatively regulate the *luxS* gene and, consequently, the synthesis of the putative quorum-sensing signaling molecule, autoinducer 2 (AI-2) (16–18). Other classes of regulators reported in C. difficile include the transcriptional regulators CcpA (19), CodY (20), and SigH (21), which influence the expression of the exotoxins, TcdA and TcdB, and Spo0A, which is a key regulator of sporulation and is important for persistence and transmission within the host (22–24). Furthermore, the C. difficile flagellar regulon modulates toxin production *in vitro* (25) in addition to having a contributory role in adhesion and colonization *in vivo* (26).

Analysis of the genome sequence of the C. difficile 027 isolate R20291 identified a locus with similarity to the *agr* operon, which is a conserved determinant in many Gram-positive bacteria (14). In Staphylococcus aureus, the global regulation of virulence genes is coordinated by the *agr* quorum-sensing locus, *agrACDB* (27–29). The *agrD* and *agrB* genes encode the precursor to the small secreted cyclic autoinducing peptide (AIP) and a transmembrane protein involved in processing and exporting of AIP, respectively. Extracellular accumulation of AIP activates a typical bacterial TCS by binding to AgrC, a sensor kinase, subsequently resulting in the phosphorylation of the AgrA response regulator. Phosphorylated AgrA binds to DNA via its C-terminal LytTR domain and can activate the transcription of both the RNAII (*agrBDCA*) transcript, creating a positive feedback loop, and the divergent RNAIII transcript encoding a regulatory RNA effector molecule (30, 31). The C. difficile agr locus carries the requisite genes for a functional *agr* operon, *agrACDB*. Interestingly, this complete locus is absent from the first reported C. difficile 630 genome, ribotype 012, and it was originally termed “*agr2*”. All analyzed C. difficile genomes contain the so-called *agr1* locus, encoding a partial *agr*-like locus of *agrDB* (14).

Originally thought to be specific to 027, a comparative genomic hybridization study identified the complete *agr* locus, *agrACDB*, in other clinical isolates, suggesting that this locus is prevalent within the species (32). Here, we undertake further analysis of C. difficile R20291 to determine the network of genes under the regulatory control of the *agr* locus. We identify a number of characteristics, including flagellar biosynthesis, TcdA production, and bis-(3′-5′)-cyclic dimeric GMP (c-di-GMP) signaling proteins that are influenced by the *agr* locus and show that it has a contributory role for colonization in the C. difficile murine model of infection.

## MATERIALS AND METHODS

### Bacterial growth conditions and strains.

All strains and plasmids used in this study are summarized in Table 1. C. difficile strains were routinely cultured at 37°C under anaerobic conditions (Mini-Mac 250; Don Whitley Scientific) using brain heart infusion (BHI; Oxoid) medium or Brazier's CCEY agar supplemented with 4% egg yolk (Bioconnections). Where appropriate, C. difficile agar was supplemented with d-cycloserine (250 μg · ml^−1^) and cefoxitin (8 μg · ml^−1^), 15 μg/ml thiamphenicol (Sigma), or 20 μg/ml lincomycin (Sigma). Escherichia coli strains were cultured aerobically at 37°C using Luria-Bertani (LB) media (Sigma). Where appropriate, media were supplemented with 12.5 μg/ml chloramphenicol (Alfa Aesar). Spore enumeration was performed by inoculating cultures 1:1 with 100% (vol/vol) ethanol for 1 h at room temperature to kill the vegetative cells. Total CFU were enumerated by serial dilution in phosphate-buffered saline (PBS) and plating on BHI plates supplemented with 0.5% taurocholate (Sigma) to stimulate germination (33).

**Table 1:** Strains and plasmids used in this study

Strain or plasmid	Characteristic(s)	Source
C. difficile strains		
R20291	Epidemic PCR ribotype 027, Stoke Mandeville hospital outbreak, United Kingdom, 2005	14
R20291 *agrA*	R20291 *agrA*76a::CT	This study
R20291 *agrA* complement	C. difficile R20291 *agrA* complemented with pMTL-84151-*agrA*	This study
E. coli CA434	Conjugation donor for E. coli HB101 [F^−^ *mcrB mrr hsdS20*(r_B_^−^ m_B_^−^) *recA13 leuB6 ara-14 proA2 lacY1 galK2 xyl-5 mtl-1 rpsL20*(Sm^r^) *glnV44* λ^−^] containing plasmid R702	41
Plasmids		
pMTL007-CE2	ClosTron plasmid (ColE1, pCD6, *catP*)	37
pMTL007-CE2-*agrA*76a	pMTL007-CE2 derivative retargeted to *agrA*	This study
pMTL-84151	E. coli-C. difficile shuttle plasmid (pCD6, *catP*, ColE1+*tra*)	42
pMTL-84151-*agrA*	pMTL-84151 containing 0.768-kb *agrA* coding sequence and 0.372-kb putative upstream promoter region	This study

### Library preparation and genomic DNA sequencing.

Genomic C. difficile DNA was isolated as previously described (34). Paired-end multiplexed libraries were created as previously described (35), and the Illumina HiSeq 2000 platform was used for whole-genome sequencing of C. difficile R20291 and R20291 *agrA*76a::CT. Sequencing reads were mapped to the R20291 genome (14) using the Burrows-Wheeler Aligner (BWA) (36).

### Mutagenesis and genetic complementation studies.

The ClosTron system was used to insertionally inactivate the R20291 *agrA* gene as previously described (37, 38). Briefly, using the Perutka algorithm (39), plasmid pMTL007C-E2 was retargeted to the antisense strand of *agrA* between positions 76 and 77, and the resultant plasmid, pMTL007C-E2-*agrA*76a, was synthesized by DNA 2.0 (Menlo Park). This plasmid was conjugated into wild-type R20291 as previously described (40) using the electrocompetent E. coli CA434 donor strain (41). Transconjugants were selected on agar plates in the presence of thiamphenicol to select for the plasmid and cycloserine/cefoxitin to select against E. coli. ClosTron integrants then were isolated by their resistance to lincomycin, and loss of the plasmid was confirmed by sensitivity to thiamphenicol. Insertions in *agrA* were confirmed by PCR screening using the primers pairs *agrA*76a-Fw/*agrA*76a-Rv, *agrA*76a-R/EBS, and RAM-Fw/RAM-Rv and sequenced using the Illumina HiSeq platform. For complementation studies, the *agrA* coding sequence and putative promoter region, 372 bp upstream, was PCR amplified from wild-type R20291 template using oligonucleotides *agrA*cNdeI_Fw and *agrA*cHindIII_Rv and inserted into the modular vector pMTL84151 by restriction and ligation cloning into the NdeI/HindIII sites (42), creating the complementation vector pMTL-84151-*agrA*. The plasmid was confirmed by DNA sequencing, and pMTL-84151-*agrA* was conjugated into C. difficile R20291 *agrA*76a::CT as described previously (40).

### RNA preparation and cDNA synthesis.

Bacterial cells were harvested in RNAprotect bacteria (Qiagen), and total RNA was extracted using the FastRNA Pro blue kit (MP Biomedical) according to the manufacturer's protocol. RNA was eluted in nuclease-free water and quantified using a NanoDrop ND-1000 and 2100 Bioanalyzer (Agilent Technologies). Genomic DNA was removed using one treatment of Turbo DNase (Applied Biosystems), and PCR analyses using primer pairs that amplified housekeeping genes *dxr*, *sigA1*, and *sigB* were performed to confirm DNA depletion. Equal amounts of total DNA-free RNA (5 μg) were reverse transcribed using random hexamer primers (Invitrogen) and SuperScript III reverse transcriptase (Invitrogen). cDNAs were synthesized in the presence of actinomycin D to prevent spurious second-strand cDNA synthesis, which inhibits DNA-dependent DNA synthesis (43).

### cDNA sequencing and expression profiling.

cDNA sequencing was performed using an Illumina HiSeq platform from 150- to 250-bp multiplexed cDNA libraries. Seventy-five cycles of paired-end sequencing from approximately 169 million cDNAs yielded 12,786 Mb of sequencing data and 15 to 45 million reads per library (see Table S1 in the supplemental material). cDNA sequence reads were aligned to the C. difficile R20291 genome reference (14) using BWA with a quality parameter (−*q*) of 15, resulting in 76 to 82% of total reads aligned per library. Reads were mapped to annotated coding sequences (CDSs) and intergenic regions to account for possible unannotated noncoding RNAs. Raw read counts were calculated per nucleotide for each gene and intergenic region from three biological replicates of wild-type R20291 and the R20291 *agrA*76a::CT mutant. Differential expression analyses were performed using R version 2.15.0 and the DESeq statistical analysis package (44). *P* values were corrected for multiple testing using the Benjamini-Hochberg method, and a *q* value threshold of 0.1 was used to define differentially regulated genes with an expected false discovery rate of <10% (see Fig. S2 in the supplemental material).

### Quantitative reverse transcription-PCR (qRT-PCR).

Relative expression levels of target transcripts were determined using Power SYBR green PCR master mix (Invitrogen) by following the manufacturer's protocol. Specific primer pairs for *tcdA*, *fliC*, CDR20291_*1514* (KEGG accession number) and the *rpoA* internal control were designed using Primer3 software (http://frodo.wi.mit.edu). RNA from three biological replicates, independent of the RNA-seq samples, was prepared from R20291 and R20291 *agrA*76a::CT isolated from late exponential growth phase. Comparative threshold cycle (*C_T_*) analysis was performed, and the mean expression from three biological replicates and three replicates of each for target transcripts was calculated (45). Mean *C_T_* values were normalized to the internal control housekeeping gene, *rpoA*. Relative mRNA expression was represented by fold change (see Fig. 3).

### Oligonucleotides.

The complete list of oligonucleotides used in this study is provided in Table S2 in the supplemental material.

### Bioinformatics.

Multiple sequence alignments were created using ClustalW2 (46, 47).

### TEM.

Negative staining and transmission electron microscopy (TEM) were performed to visualize C. difficile flagella. R20291, R20291 *agrA*76a::CT, and *agrA* complement strains were grown under the same conditions as samples prepared for RNA processing and TcdA quantification. Cultured colonies were mixed with distilled water to create a slightly turbid suspension and applied to Formvar/carbon-coated EM grids. An equal volume of 3% ammonium molybdate with 1% trehalose was added to negative stain. Images were taken on an FEI Spirit Biotwin 120-kV TEM with a Tietz F415 charge-coupled-device (CCD) camera.

### TcdA quantification.

C. difficile cultures were grown in BHI broth with shaking to late exponential phase, and culture supernatants were removed. TcdA quantification was performed by sandwich enzyme-linked immunosorbent assay (ELISA) as previously described (23). CFU were determined to ensure equal numbers of vegetative cells in all samples and replicates.

### CI experiments.

The C. difficile murine model of infection was used to perform competitive index (CI) experiments as previously described (23). Wild-type C57BL/6 mice (*n* = 5) were infected with 5 × 10^6^ spores via gavage in 0.2 ml PBS. Equal amounts of spores from the parental R20291 and isogenic R20291 *agrA*76a::CT mutant derivative were used. Fecal samples were collected and enumerated by plating on C. difficile CCYE agar, with and without lincomycin, and incubated for 48 h. Agar supplemented with lincomycin selected for the knockout containing the *ermB* cassette. The CI number was determined using the following ratio: (R20291 *agrA*76a::CT/R20291 wild-type)_output_/(R20291 *agrA*76a::CT/R20291 wild-type)_input_. Statistical testing was performed using the Mann-Whitney test applied to log_10_ values of the CI ratios. All animal infections were performed in accordance with the United Kingdom Home Office Animals (Scientific Procedures) Act of 1986.

### RNA-seq data accession number.

RNA-seq data generated in this work are available online at ArrayExpress under accession number E-ERAD-97.

## RESULTS

### The distribution of the C. difficile agr locus.

It has previously been shown that the *agr* locus is absent from some C. difficile isolates (14, 32). To better understand the prevalence of *agr* within the C. difficile species, we carried out comparative genomic analysis to determine its distribution within sequenced and fully annotated C. difficile isolates (Table 2) (14, 48). This revealed that the *agr* locus is not limited to the ribotype 027 strains but is present in multiple disease-causing C. difficile lineages, including PCR ribotypes 001 and 017.

**Table 2:** Distribution of *agr* locus in fully annotated C. difficile genomes

Strain and presence of *agr* locus	Ribotype	Source	Year and source of origin	Reference	Accession no.
*agr* locus positive					
BI-9	001	Human	2001, United States	48	FN668944
M68	017	Human	2006, Ireland	48	FN668375
CF5	017	Human	1995, Belgium	48	FN665652
BI-1	027	Human	1988, United States	48	NC_017179
2007855	027	Bovine	2007, United States	48	FN665654
R20291	027	Human	2006, United Kingdom	14	NC_013316
CD196	027	Human	1985, France	14	NC_013315
*agr* locus negative					
630	012	Human	1982, Switzerland	16	AM180355
M120	078	Human	2007, United Kingdom	48	FN665653

**Fig 1:**
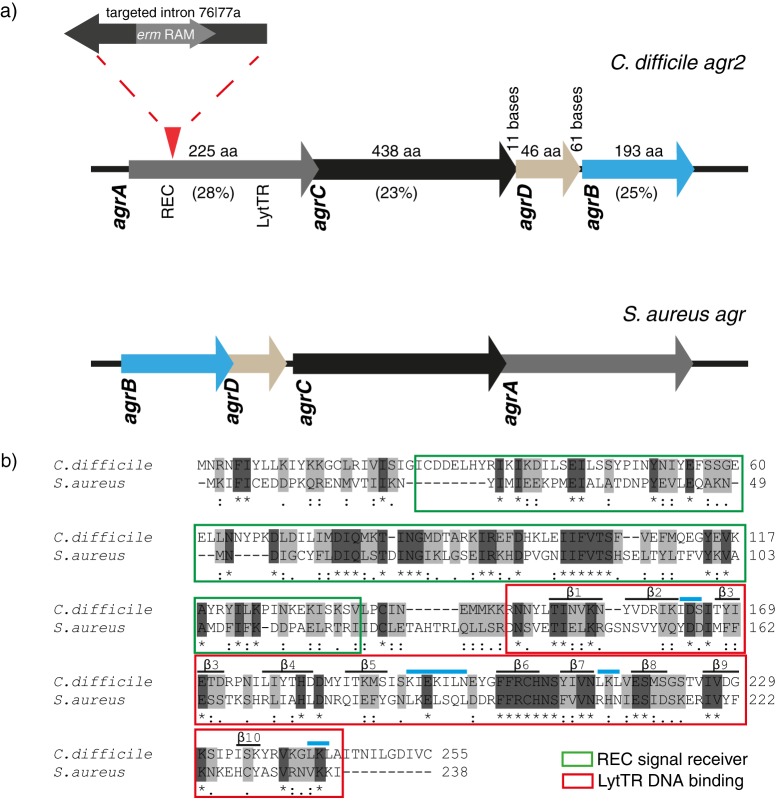
Genetic organization of the C. difficile 027 *agr* locus. (a) The complete R20291 *agr* locus (upper panel) compared to the *agr* locus of S. aureus. Arrows indicate genes and direction of transcription. The length of each protein is indicated above (aa, amino acids), and the percent identity to the respective S. aureus orthologue is indicated below. The location of the group II intron target site, according to the ClosTron delivery system, used to construct the R20291 *agrA*76a::CT mutant strain is shown. (b) ClustalW2 multiple sequence alignment of AgrA from C. difficile and S. aureus. Symbols: *, identical amino acids; :, strongly similar; ., weakly similar. AgrA shares 28% amino acid identities, as determined by BLAST search. The two domains of AgrA are outlined: 5′ REC signal receiver domain, green box; 3′ LytTR-DNA binding domain, red box. The structure of the S. aureus AgrA LytTR-DNA binding domain (residues 137 to 238) is indicated (72). The secondary structure is indicated: helices, blue bars; strands, black bars.

Analysis of the genome of C. difficile R20291 revealed that the *agr* locus included *agrA*, *agrC*, *agrD*, and *agrB*, respectively (14). Interestingly, in the S. aureus agr operon these genes are in the reverse order (*agrBDCA*) (Fig. 1a). The C. difficile R20291 *agrA*-encoded protein shares 28% amino acid identity with the equivalent S. aureus AgrA and contains both a predicted N-terminal REC signal receiver domain and C-terminal LytTR-DNA binding domain (Fig. 1b). The C. difficile R20291 *agrC*-encoded protein and *agrB*-encoded proteins share 23 and 25% amino acid identity, respectively, with the equivalent S. aureus orthologues. The putative 46-amino-acid C. difficile R20291 AgrD polypeptide shares no significant similarity with S. aureus. This is consistent with the highly variable nature of this peptide observed among S. aureus isolates (49). A RNAIII divergent transcript has not been identified in C. difficile.

To determine if the C. difficile R20291 *agr* region is transcribed when grown under standard laboratory conditions, RT-PCR analysis of R20291 was performed using primer pairs specific to the C. difficile R20291 *agrACDB* coding sequences. This confirmed that the full locus is expressed at exponential (optical density at 600 nm [OD_600_] = 0.3) and late exponential (OD_600_ = 0.7) phase in BHI media.

### Insertional inactivation of C. difficile R20291 *agrA*.

To study the role of the *agr* locus in C. difficile R20291, an isogenic mutant of *agrA* was constructed using the ClosTron system (37). The genotype of the C. difficile R20291 *agrA*76a::CT mutant derivative was confirmed by PCR analysis exploiting specific primers (data not shown). Illumina sequencing of whole-genome DNA purified from the C. difficile R20291 *agrA*76a::CT mutant derivative revealed that no secondary mutations were acquired, and the genetic background was otherwise identical to the parental R20291 aside from the anticipated single intron insertion. Characterization of the growth kinetics of C. difficile R20291 *agrA*76a::CT and R20291 revealed no obvious differences under the conditions tested (see Fig. S1 in the supplemental material).

### RNA-seq analysis of the regulon in the C. difficile R20291 *agrA*76a::CT mutant.

**Fig 2:**
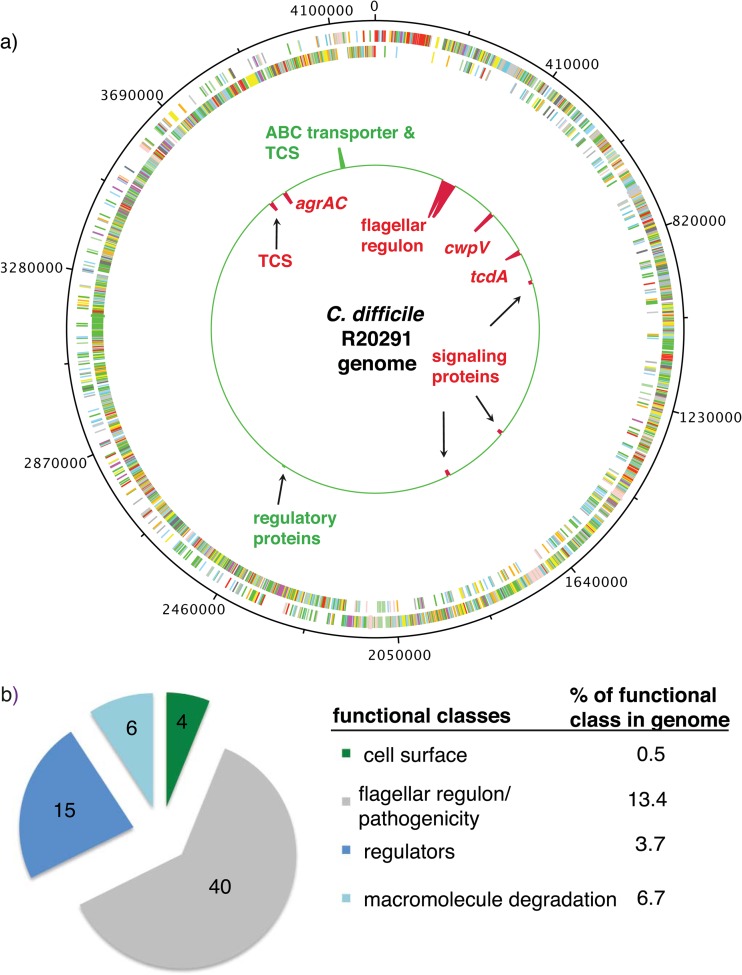
Chromosomal representation of R20291 *agrA*76a::CT differentially expressed genes. (a) The log_2_ fold change of differentially expressed transcripts mapped per base pair to C. difficile R20291 whole-genome sequence in DNA plotter (73). The outermost circles represent the CDSs of the forward strand (first circle) and the reverse strand (second circle) colored by functional classes assigned. The inner circle represents differentially expressed transcripts (*P* ≤ 0.1) (red peaks, underexpressed; green peaks, overexpressed). (b) Categorization of differentially expressed transcripts according to functional classes. The percentages of each functional class represented relative to the whole genome are listed. Three functional classes were enriched relative to background distributions: flagellar regulon and pathogenicity (*P* = 6.38 × 10^−22^), regulators (*P* = 0.002), and macromolecule degradation (*P* = 0.001) (*P* ≤ 0.05 by hypergeometric test).

**Fig 3:**
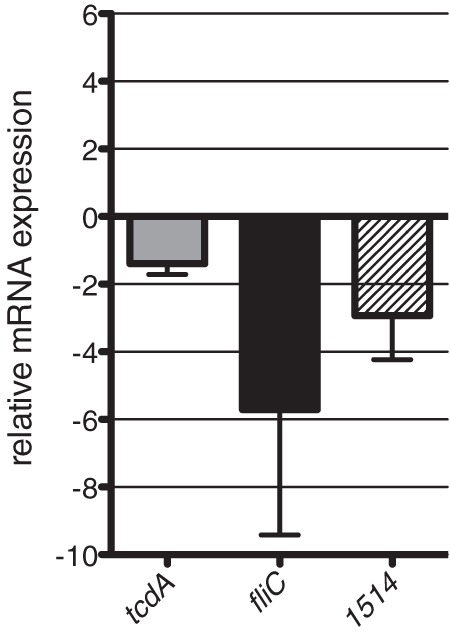
Validation of RNA-seq by quantitative real-time PCR analysis. Relative mRNA levels of transcripts corresponding to *tcdA*, *fliC*, and CDR20291_*1514*. Data are from three independent experiments performed in triplicate, and error bars indicate standard deviations. Fold change of R20291 *agrA*76a::CT mutant mRNA levels are indicated relative to wild-type R20291 levels.

As the S. aureus agr locus plays a key role in controlling the coordinated expression of virulence genes, we used RNA-seq analysis to begin to define the regulon under the control of the C. difficile R20291 *agr* locus. To this end, total RNA was isolated from C. difficile R20291 and R20291 *agrA*76a::CT grown to late exponential phase. The log_2_ fold change of transcript abundance between these RNA populations was obtained (*P* ≤ 0.1) (see Fig. S2 in the supplemental material), and transcripts were visualized by mapping the coverage per base pair to the reference R20291 genome (Fig. 2a). In total, 75 transcripts were found to be significantly differentially expressed in the R20291 *agrA*76a::CT mutant. The differentially regulated transcripts were categorized according to their functional class, and the enrichment of each represented class was determined. The functional classes of flagellar regulon and pathogenicity, regulators, and macromolecule degradation were enriched for differentially expressed genes in R20291 *agrA*76a::CT compared to R20291 (*P* ≤ 0.05 by hypergeometric test) (Fig. 2b). Validation of RNA sequencing results was performed by qRT-PCR. In agreement with the R20291 *agrA*76a::CT transcriptome, *tcdA*, *fliC*, and CDR20291_*1514* transcripts all were underrepresented (−1.39-, −5.64-, and −2.93-fold change, respectively) compared to wild-type R20291 (Fig. 3).

The transcript of *agrC*, encoding the cognate sensor kinase directly downstream of *agrA*, was underexpressed in the R20291 *agrA*76a::CT transcriptome (*P* = 2.17 × 10^−18^). There may be a regulatory role of AgrA in *agrC* expression causing this decrease. Alternatively, it is possible that the ClosTron insertion in the *agrA* gene affects the transcription of the subsequent gene in the locus, in this case *agrC*. Polar effects resulting from insertional inactivation using the ClosTron system have been described previously in independent mutants located in the C. difficile flagellar region (*fliF*, *fliG*, and *flhB-flhR*), which resulted in 8- to 20-fold downregulation of the downstream transcript, *fliA* (25, 50).

Of the 75 differentially expressed transcripts, 64 were determined to be underrepresented in the R20291 *agrA*76a::CT transcriptome (see Table S3 in the supplemental material). Most strikingly, the transcripts of the majority of genes involved in flagellum formation were underexpressed. Of the 54 genes believed to be involved in flagellum formation, 50 were affected, including all of the regulatory and structural genes, with the exception of *fliN*, encoding the hypothetical C-ring protein. The flagellin, the major structural protein of flagella, encoded by *fliC*, was underexpressed 6.5-fold (*P* = 2.05 × 10^−20^). The anti-sigma factor encoded by *flgM* and alternative sigma factor encoded by *fliA* were underrepresented 6.2- and 4.2-fold, respectively, and may be direct targets of the transcriptional regulator AgrA. Three of the six flagellin modification genes found in the 027 strains were also found to be underexpressed, including two putative glycosyl transferases, CDR20291_*0242* and CDR20291_*0243* (3.7- and 2.7-fold change, respectively).

Expression of mRNA encoding TcdA was underexpressed 2.4-fold (*P* = 3.0 × 10^−4^) in R20291 *agrA*76a::CT compared to that in R20291 (see Table S3 in the supplemental material). The reason for this altered expression cannot be determined from these data, but it is interesting that the C. difficile flagellar regulon has been reported recently to modulate toxin A production (25). TcdB was not as highly expressed as TcdA in the R20291 wild-type and R20291 *agrA*76a::CT mutant transcriptomes and was not differentially regulated in the R20291 *agrA*76a::CT transcriptome.

Ten genes annotated as regulatory proteins were differentially expressed in R20291 *agrA*76a::CT compared to wild-type R20291. These included three genes linked to c-di-GMP signaling, CDR20291_*0685*, CDR20291_*1268*, and CDR20291_*1514*, which were underrepresented in R20291 *agrA*76a::CT and encode proteins that contain GGDEF or EAL domains (see Table S3 in the supplemental material). Furthermore, three transcripts encoding putative TCSs, CDR20291_*3126* to CDR20291_*3128*, were also underexpressed in the R20291 *agrA*76a::CT transcriptome (see Table S3 in the supplemental material).

C. difficile is predicted to contain 234 putative small noncoding RNAs (51). In this study, 10 intergenic regions were differentially regulated in R20291 *agrA*76a::CT (see Tables S3 and S4 in the supplemental material). Each intergenic sequence was searched against a nonredundant nucleotide collection using NCBI BLAST, but no evidence of sequence conservation outside the Clostridium genus was found. Additionally, each sequence was searched against the Rfam database (52), but no Rfam matches were identified for 9/10 differentially regulated intergenic regions. However, the 5′ untranslated intergenic region upstream of the flagellar operon where the C. difficile c-di-GMP riboswitch, Cd1, is located was underexpressed 5.3-fold (*P* = 1.22 × 10^−13^). Due to its location, Cd1 is suggested to regulate flagellar biosynthesis genes and motility by responding to cyclic di-GMP concentrations (53).

### *agr* locus positively regulates C. difficile flagellar biosynthesis and TcdA *in vitro*.

**Fig 4:**
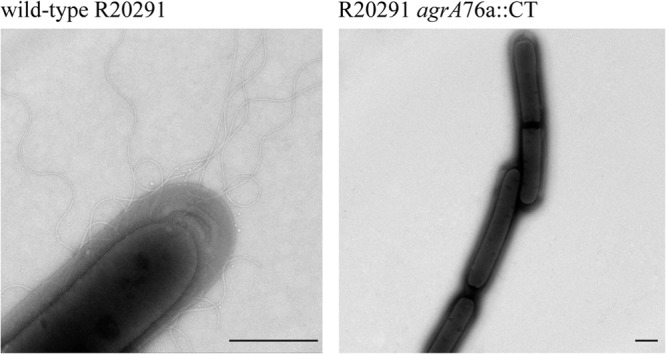
Visualization of C. difficile flagellar filaments. Electron microscopy reveals the absence of flagella in R20291 *agrA*76a::CT. Cultures were grown to late exponential phase in BHI broth. Scale bar, 1 μm.

Based on the RNA-seq transcriptome data, we hypothesized that the C. difficile R20291 *agrA*76a::CT mutant would be unable to form flagellar filaments. Consequently, cultures of R20291 and R20291 *agrA*76a::CT were prepared as described for the RNA-sequencing analysis and examined using electron microscopy for the formation of flagella. This analysis revealed that while peritrichous flagellar filaments were abundant on the cell surface of R20291, equivalent structures were absent from similarly treated R20291 *agrA*76a::CT cultures (Fig. 4).

**Fig 5:**
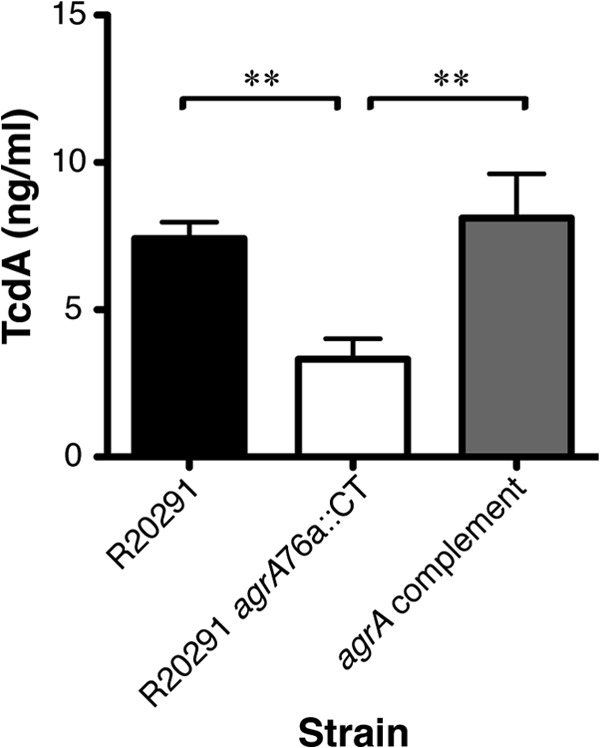
*agr* locus positively regulates TcdA levels. Relative amounts of TcdA produced by C. difficile R20291, R20291 *agrA*76a::CT, and *agrA* complement grown to late exponential phase in BHI broth are shown. C. difficile R20291 produced TcdA at 7.42 ng/ml, the *agrA* strain produced it at 3.32 ng/ml, and the *agrA* complemented strain produced it at 8.11 ng/ml. Data represent four independent experiments performed in triplicate. Error bars represent standard errors of the means. Asterisks indicate statistically significant differences (**, *P* < 0.01 by Student's *t* test).

A sandwich ELISA was used to determine the levels of TcdA expressed in R20291 or R20291 *agrA*76a::CT culture supernatants grown under conditions equivalent to those used for RNA preparation (Fig. 5). C. difficile R20291 cultures harbored detectable TcdA at 7.42 ng/ml, which is comparable to R20291 *agrA*76a::CT at 3.32 ng/ml when cultured under equivalent conditions.

The *agrA* complementation vector (pMTL-84151-*agrA*) was introduced into the R20291 *agrA*76a::CT mutant to confirm that the observed phenotypes were the result of the ClosTron insertion in the *agrA* gene (see Materials and Methods). Growth kinetics for wild-type R20291, R20291 *agrA*76a::CT, and *agrA* complemented strains revealed similar growth dynamics between all three strains (see Fig. S1 in the supplemental material). The *agrA* complementation vector did not restore the flagellar filaments to the cell surface in the R20291 *agrA*76a::CT mutant strain. It is possible that the complementation vector did not express wild-type levels of *agrA*; therefore, it failed to complement the flagella. However, the *agrA* complemented strain successfully restored TcdA production to 8.11 ng/ml, comparable to wild-type R20291 levels (*P* = 0.008) (Fig. 5).

### C. difficile R20291 *agrA*76a::CT exhibits an early colonization defect in mice.

**Fig 6:**
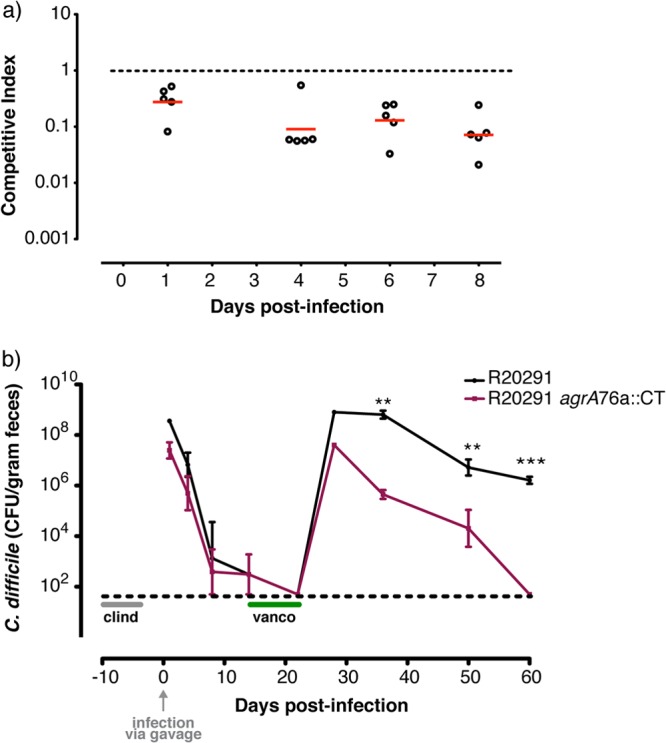
C. difficile R20291 *agr* locus mediates efficient colonization and relapsing infection in the murine model of infection. Mice were treated with clindamycin (clind; represented as a gray line) for 7 days prior to infection via gavage with equal amounts of C. difficile R20291 and R20291 *agrA*76a::CT. Fecal shedding of R20291 and the R20291 *agrA*76a::CT mutant was monitored. Data are representative of two independent experiments (*n* = 5 per group). (a) Competitive index (CI) course of R20291 and R20291 *agrA*76a::CT mutant monitored over 8 days. Open circles indicate CI values from individual mice, and the red horizontal bars represent the geometric means. R20291 *agrA*76a::CT mutant is attenuated at 1, 4, 6, and 8 days postinfection as determined by Mann-Whitney test. (b) To test whether the mutation in the R20291 *agrA* locus influences relapsing infection, mice received a 7-day course of vancomycin (vanco; represented as a green line) following the initial infection, during which fecal shedding of C. difficile decreased to below the detection limit (represented as a dashed horizontal line). Fecal shedding of C. difficile relapsed in both groups; however, R20291 *agrA*76a::CT levels were consistently reduced. Statistically significant differences are indicated by asterisks (*, *P* < 0.05; **, *P* < 0.01; ***, *P* < 0.001) and were determined by two-way analysis of variance.

C. difficile R20291 can establish a chronic intestinal infection in the murine model, with relapse infection consistently occurring after cessation of vancomycin treatment (54–56). To assess any role of the *agr* locus in the murine model of infection, direct fitness comparisons using competitive index (CI) experiments were performed. Here, healthy C57BL/6 mice (*n* = 5 per group) were orally infected after clindamycin treatment with equal numbers of viable R20291 and R20291 *agrA*76a::CT spores (5 × 10^6^ CFU), and C. difficile CFU in fecal sheddings were monitored for 8 days after the challenge. C. difficile R20291 *agrA*76a::CT was shed at significantly lower levels than R20291 at 1, 4, 6, and 8 days postchallenge (Fig. 6a). To determine if the *agr* locus influences relapse, the mice from similar CI challenges were treated with a clinically relevant dose of oral vancomycin for 7 days (Fig. 6b), and C. difficile fecal shedding was again monitored. Fecal shedding of C. difficile returned in both groups within 3 days of vancomycin cessation. However, while the R20291 fecal shedding levels returned to pre-vancomycin-treatment levels, those of R20291 *agrA*76a::CT were consistently reduced. Statistical analysis confirmed that R20291 *agrA*76a::CT is relatively attenuated in terms of relapse infection.

## DISCUSSION

Previous whole-genome sequencing analysis of the epidemic 027, R20291, identified the C. difficile agr locus (*agrACDB*), which showed similarity to the *agr* genes of S. aureus. Comparative genomic analysis with disease-causing ribotypes 017 and 001 confirmed that this locus is not specific to ribotype 027, as previously thought (48). The *agr* genes of C. difficile are in the reverse order from the *agr* genes of S. aureus, while the *agr*-like genes of Enterococcus faecalis (*fsrABDC*) are also different (57). The genetic organization of *agr* genes varies between different species (57, 58). This work aimed to identify the role of this locus in the virulence of C. difficile R20291; therefore, we generated an insertion mutant in the transcriptional regulator, AgrA.

In this study, we report the first RNA sequencing analysis of a C. difficile strain to investigate the regulatory network controlled by the *agr* locus. Similar to other studies reporting transcriptional profiling of relevant *agr* loci, we investigated the regulon in the 027 *agrA* mutant when grown to late exponential phase (59). Consequently, we identified 75 genes that were differentially regulated in the R20291 *agrA*76a::CT mutant transcriptome. The genes positively and negatively regulated by the *agr* locus included flagellar biosynthesis genes, *tcdA*, c-di-GMP regulatory protein genes, and uncharacterized two-component regulatory systems.

The majority of the flagellar biosynthesis genes were underexpressed in the C. difficile R20291 *agrA*76a::CT transcriptome. This could explain the inability of the R20291 *agrA*76a::CT mutant to produce cell surface-anchored filaments *in vitro* as observed by TEM analysis. Experimental investigations have shown that insertional inactivation of the major flagellin subunit, FliC, results in the inability to produce flagellar filaments, resulting in a nonmotile phenotype and 10-fold less adherence to the murine cecum layer (60) (26). Interestingly, recent studies have shown that the C. difficile flagellar regulon has a role in the modulation of toxin A production (25). We have shown that the *tcdA* transcript is underexpressed in the R20291 *agrA*76a::CT mutant, and reduced levels of TcdA were produced *in vitro* compared to those of the parental wild-type strain, R20291. The reduced TcdA phenotype may relate to the differential expression of the flagellar regulon.

The small-molecule bacterial secondary messenger bis-(3′-5′)-cyclic dimeric GMP (c-di-GMP) is an important signaling molecule in bacteria, mediating the transition between sessile and motility lifestyles (61). C. difficile R20291 is predicted to encode up to 31 proteins involved in c-di-GMP turnover (62). C-di-GMP is synthesized by diguanylate cyclase enzymes that contain GGDEF domains, while phosphodiesterases that contain EAL or HD-GYP domains stimulate degradation of the messenger (63). The three underexpressed c-di-GMP signaling proteins (CDR20291_0685, CDR20291_1268, and CDR20291_1514) in the R20291 *agrA*76a::CT mutant transcriptome are solely phosphodiesterase enzymes due to a predicted catalytically inactive GGDEF domain. The protein orthologues have been experimentally characterized in C. difficile strain 630 (CD0757, CD1421, and CD1616, respectively) (62). Purified CD0757 was confirmed to have enzymatic activity when overexpressed in Vibrio cholerae, resulting in enhanced motility on soft agar (62). Conversely, mutating the glutamic acid residue of the EVLxR motif, important for enzymatic activity of the phosphodiesterase, abolished this enhanced motility phenotype (62). The *agr*-regulated c-di-GMP EAL-containing proteins, encoded by CD1421 and CD1616 in strain 630, also exhibited enhanced motility phenotype when overexpressed in V. cholerae. In agreement, Purcell and colleagues demonstrated that artificial elevation of c-di-GMP levels negatively regulated motility and flagellar biosynthesis genes (*flgB*, *fliA*, and *flgM*) in C. difficile 630 (64). Furthermore, our data reveal the differential expression of novel C. difficile transcriptional regulators and two-component systems, such as the sensor histidine kinase and cognate response regulator encoded by CDR20291_*3424* and CDR20291_*3425*, respectively. Transcriptional regulators are commonly associated with the signaling network of c-di-GMP; in Salmonella enterica serovar Typhimurium, the response regulator CsgD activates the expression of diguanylate cyclase-containing protein, AdrA, in turn triggering cellulose biosynthesis (65, 66). It is possible that the levels of c-di-GMP in the R20291 *agrA*76a::CT mutant are increased due to the underexpression of the EAL-containing regulatory proteins, which stimulate degradation of the small cyclic molecule. The elevated c-di-GMP levels may be negatively affecting the flagellar biosynthesis genes, similar to the findings from Purcell et al. (64).

In addition to S. aureus, orthologous *agr* systems have been shown to be relevant for virulence in pathogenic firmicute species (57, 67). The inactivation of the *agrA* gene of L. monocytogenes attenuates virulence of the bacterium in the murine model, causing a 50% lethal dose (LD_50_) 10-fold higher than that of the wild-type strain (67). Furthermore, isogenic mutant strains of the *agr*-like locus in E. faecalis were attenuated for virulence in the rabbit endophthalmitis model (68, 69), in the nematode Caenorhabditis elegans model (70), and in the mouse peritonitis model (57). Here, we have demonstrated that the 027 *agr* locus contributes to colonization and relapsing infection in the C. difficile murine infection model. The mechanism by which the R20291 *agrA*76a::CT mutant has reduced colonization and reduced relapse infection is difficult to speculate, as the mutation affects 75 genes *in vitro*. The inability of the R20291 *agrA*76a::CT mutant to form flagellar filaments may contribute to the attenuated colonization and relapse infection observed; similarly, TcdA may be necessary for efficient colonization of C. difficile R20291 in the murine model. A direct comparison in the murine infection model between defined *agrA*, *tcdA*, and aflagellate mutants may help to define the relative contribution of these determinants in colonization.

Whole-genome sequencing analysis of the R20291 *agrA*76a::CT mutant confirmed that the observed phenotypic differences were due solely to the insertional inactivation of the *agrA* gene and not acquired secondary mutations. The variation in the effectiveness of complementation may result from relatively low expression of AgrA from the complementing plasmid, allowing AgrA to bind only to the highest affinity sites. Furthermore, polar effects resulting from the AgrA mutation in downstream coding sequences may have inhibited effective complementation studies. The insertional deletion of *agrA* resulted in the underexpression of the downstream coding region of *agrC*, suggesting that *agrAC* form a single transcriptional unit. Similarly, the C. acteobutylicum agr cluster is also predicted to comprise two transcriptional units, *agrBD* and *agrCA* (71). However, the complete C. difficile agr locus is significantly underrepresented in the R20291 *agrA*76a::CT mutant at exponential phase in BHI broth (data not shown), suggesting that *agrACDB* is a single transcript, similar to S. aureus agr RNAII.

In conclusion, we demonstrate that the *agr* locus modulates known C. difficile virulence factors *in vitro* and has a contributory role in colonization and relapse of epidemic C. difficile 027 *in vivo*. We propose that this is due to the transcriptional regulatory control of the *agr* locus and have demonstrated its effect on the expression of multiple determinants by RNA sequencing.

## Supplementary Material

Supplemental material
