# Supplementary material for: The agr Locus Regulates Virulence and Colonization Genes in Clostridium difficile 027
Source: J Bacteriol. 2013 Aug;195(16):3672–81. doi: 10.1128/JB.00473-13 (PMC3754575; doi:10.1128/JB.00473-13)
Supplement: Supplemental material [file supp_195_16_3672__index.html]

Supplemental material 

# The *agr* Locus Regulates Virulence and Colonization Genes in Clostridium difficile 027

## Supplemental material

**Files in this Data Supplement:**

- Supplemental file 1 -

  Fig. S1, growth kinetics of wild-type R20291, R20291 *agrA76*a::CT, and *agrA* complement strains

  Fig. S2, scatter plot of log2 fold change versus mean expression

  Table S1, mapping statistics for RNA-seq libraries

  Table S2, oligonucleotides

  Table S3, downregulated transcripts in R20291 *agrA76*a::CT mutant

  Table S4, upregulated transcripts in R20291 *agrA76*a::CT mutant

  PDF, 554K
