# Supplementary material for: The agr Locus Regulates Virulence and Colonization Genes in Clostridium difficile 027
Source: J Bacteriol. 2013 Aug;195(16):3672–81. doi: 10.1128/JB.00473-13 (PMC3754575; doi:10.1128/JB.00473-13)
Supplement: Supplemental material [file JB.00473-13_zjb999092730so1.pdf]

Supplementary Information

The *agr* locus regulates virulence and colonization genes in *Clostridium difficile* 027

Melissa J. Martin, Simon Clare, David Goulding, Alexandra Faulds-Pain, Lars Barquist, Hilary P. Browne, Laura Pettit, Trevor D. Lawley, Gordon Dougan, Brendan W. Wren

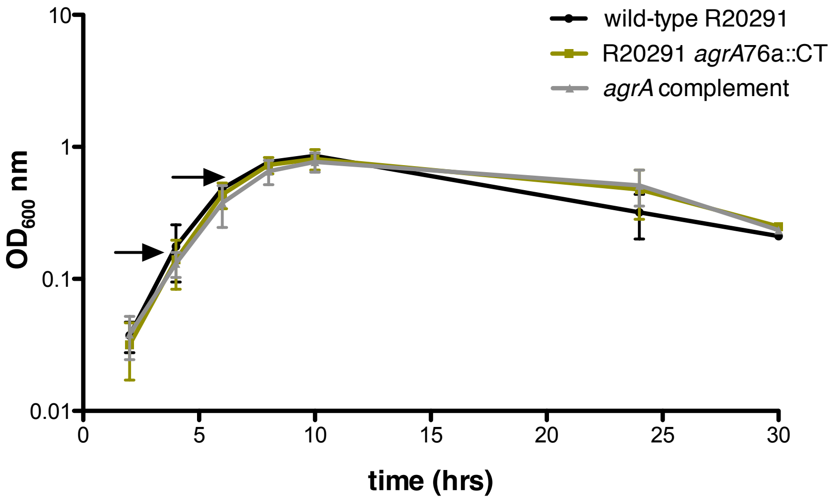

Supplementary Figure 1 Growth kinetics of wild-type R20291, R20291 *agrA*76a::CT, and *agrA* complement strains. *C. difficile* cultures were grown in BHI media with shaking under anaerobic conditions. Samples were taken every two hours and the optical density was determined. Arrows indicate exponential and late exponential times points where RNA was extracted for expression analysis of R20291 and R20291 *agrA*76a::CT strains.

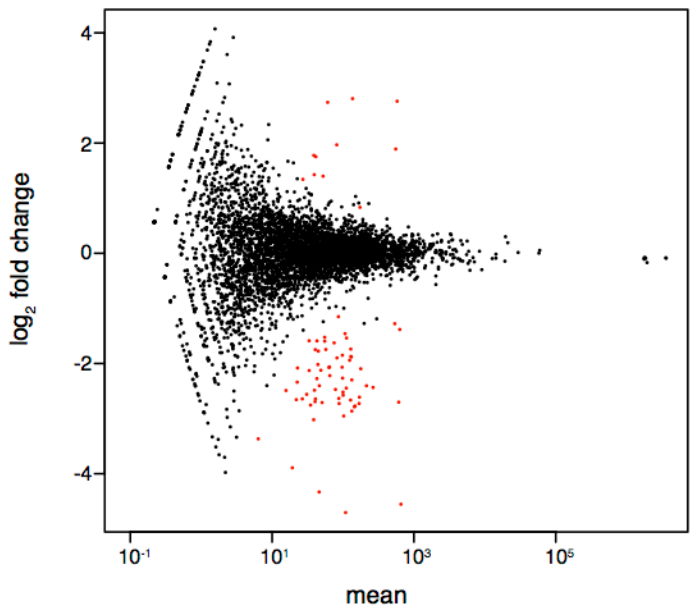

Supplementary Figure 2 Scatter plot of $\log_2$ fold change versus mean expression. Red color indicates genes differentially expressed in the R20291 *agrA*76a::CT mutant at a false discovery rate of 10% ($p < 0.1$ after applying the Benjamini-Hochberg multiple testing).

Supplementary Table 1 Mapping statistics for RNA-seq libraries

Sample name	Total number of reads	Reads mapped to genome	Total number of bases mapped (Mb)	Minimum coverage depth (%)	Mean coverage depth
R20291_1	15,628,448	12,550,972 (80.3%)	938.32	45.0	17.93x
R20291_2	44,795,394	35,791,067 (79.9%)	3440.64	69.0	34.75x
R20291_3	15,587,834	12,423,250 (79.7%)	1198.08	53.0	20.99x
R20291 *agrA*76a::CT _1	27,657,376	21,600,070 (78.1%)	2119.68	62.6	28.6x
R20291 *agrA*76a::CT _2	45,402,726	37,425,121 (82.4%)	3491.84	62.2	29.16x
R20291 *agrA*76a::CT _3	20,750,232	15,799,460 (76.1%)	1597.44	60.8	26.12x

Supplementary Table 2 Oligonucleotides used in this study

**Oligonucleotide**	**Sequence (5' →3')**
Intron integration screening:	
*agrA*76a-Fw	TTCATTTGTATGTCCATAATTAATATGTC
*agrA*76a-Rv	AAGAAAGGATGTTTAAGAATTGTGATTAG
RAM-Fw	ACGCGTTATATTGATAAAAATAATAATAGTGGG
RAM-Rv	ACGCGTGCGACTCATAGAATTATTTCCTCCCG
EBS universal	CGAAATTAGAAACTTGCGTTCAGTAAAC
Complementation:	
*agrA*cNdeI-Fw	ATATTTCATATGACTAACTTAATATATTTTCAGG
*agrA*cHindIII-Rv	ATATTTAAGCTT TTAGCATACTATATCCCC
RNA quality control:	
*dxr*_Fw	GCTACTTTCCATTCTATCTG
*dxr*_Rv	CCAACTCTTTGTGCTATAAA
*sigB*_Fw	CCAGCTTTGCAACACCAACT
*sigB*_Rv	CCATAAGAAGCCTCCATAGCC
*sigA1*_Fw	GATGCAGAGGCAATTTCACA
*sigA1*_Rv	CCTTCGTGCATCCTTCTAGC
RT-PCR:	
*rpoA*_Fw	GGATGATATGATGAAGGTTAGAAACCT
*rpoA*_Rv	CCCAATCCAAGTTCTTCTAGTTTTTG
*fliC*_Fw	GTGCTTTGATAGCAAATAACCAAAT
*fliC*_Rv	GAGCTCTCATTTTCTCAGATATAGCA
*tcdA*_Fw	GTCGGATTGCAAGTAATTGACAATA
*tcdA*_Rv	TAACAGTCTGCCAACCTTTTGAGA
1514_Fw	TGGGAATGAAATTTTGCGGTA
1514_Rv	CCGCAGATATTCTTGCAAAC

Supplementary Table 3 List of down-regulated transcripts in R20291 *agrA*76a::CT mutant

CDS	Product	Fold Change	log_2 fold change	pvalue-adj
**Flagellar biosynthesis**				
CDR20291_*0224*	glucose-1-phosphate thymidylyltransferase	2.9	-1.5	0.0048
CDR20291_*0225*	dtdp-4-dehydrorhamnose 3,5-epimerase	3.0	-1.6	0.0056
CDR20291_*0226*	dtdp-glucose 4,6-dehydratase	3.8	-1.9	6.40E-07
CDR20291_*0227*	putative transglycosylase	3.6	-1.9	1.84E-06
CDR20291_*0228*	conserved hypothetical protein	3.1	-1.6	7.83E-05
flgM	negative regulator of flagellin synthesis (anti-sigma-d factor)	6.2	-2.6	8.74E-06
flgN	putative flagellar biosynthesis protein	6.5	-2.7	5.55E-08
flgK	putative flagellar hook-associated protein	7.7	-3.0	4.43E-15
flgL	flagellar hook-associated protein	6.2	-2.6	6.40E-12
fliW	flagellar assembly factor	8.1	-3.0	1.54E-08
csrA	carbon storage regulator	6.3	-2.7	7.50E-05
fliS1	flagellar protein	5.9	-2.6	8.45E-07
fliS2	flagellar protein	5.1	-2.3	0.0004
fliD	flagellar cap protein	6.9	-2.8	5.77E-16
CDR20291_*0239*	conserved hypothetical protein	6.5	-2.7	1.01E-08
fliC	flagellin subunit	6.5	-2.7	2.05E-20
CDR20291_*0241*	putative glycosyltransferase	6.3	-2.7	7.62E-14

CDR20291_*0242*	glycosyl transferase	3.7	-1.9	6.66E-07
CDR20291_*0243*	glycosyl transferase	2.7	-1.5	0.0010
CDR20291_*0245*	putative carbamoyl-phosphate-synthetase	2.2	-1.2	0.0497
flgB	flagellar basal-body rod protein	4.4	-2.1	1.37E-06
flgC	flagellar basal-body rod protein	5.6	-2.5	8.29E-07
fliE	flagellar hook-basal body complex protein	6.8	-2.8	9.54E-08
fliF	flagellar M-ring protein	6.8	-2.8	7.38E-18
fliG	flagellar motor switch protein	4.9	-2.3	3.22E-12
fliH	flagellar assembly protein	2.9	-1.5	0.0001
fliI	flagellum-specific ATP synthase	6.1	-2.6	6.33E-17
fliJ	flagellar protein	4.4	-2.1	2.25E-05
fliK	putative flagellar hook-length control protein	6.0	-2.6	8.46E-13
flgD	putative basal-body rod modification protein	5.7	-2.5	2.55E-13
flgE	flagellar hook protein	7.3	-2.9	7.91E-18
flbD	flagellar protein	5.3	-2.4	1.57E-07
motA	chemotaxis protein	6.6	-2.7	4.98E-17
motB	chemotaxis protein	4.2	-2.1	3.17E-08
fliL	flagellar basal body-associated protein	4.7	-2.2	5.01E-08
fliO/fliZ	FliO/Fliz putative flagellar protein	5.5	-2.5	1.74E-10
fliP	flagellar biosynthetic protein	5.5	-2.4	3.99E-12

fliQ	flagellar export protein	6.2	-2.6	1.85E-07
fliR/flhB	flagellar export protein	5.4	-2.4	4.71E-15
flhA	flagellar export protein	4.3	-2.1	2.79E-11
flnF	signal recognition particle complex, GTP- binding subunit	4.1	-2.0	2.03E-05
fliG	flagellar number regulator	4.8	-2.3	8.15E-10
fliA	RNA polymerase sigma factor for flagellar operon	4.2	-2.1	2.27E-06
CDR20291_*0271*	putative exported protein	4.8	-2.3	2.82E-06
*flgG*1	flagellar basal-body rod protein	3.4	-1.7	5.40E-05
flgG	putative flagellar basal-body rod protein	3.0	-1.6	0.00067
fliM	putative flagellar motor switch protein	4.2	-2.1	1.23E-07
fliN	putative flagellar motor switch protein	3.7	-1.9	8.41E-06
CDR20291_*0276*	conserved hypothetical protein	3.7	-1.9	4.03E-07
CDR20291_*0440*	cell surface protein (putative hemagglutinin/adhesin)	23.5	-4.6	1.85E-57
**Toxin**				
tcdA	toxin A	2.4	-1.3	0.0003
**Signaling proteins**				
CDR20291_*0685*	ci-di-GMP protein	3.4	-1.8	2.79E-05
CDR20291_*1268*	ci-di-GMP protein	2.6	-1.4	0.0713
CDR20291_*1514*	putative signaling protein	6.6	-2.7	1.74E-11
CDR20291_*3126*	two-component response regulator	5.6	-2.5	0.0301

CDR20291_*3127*	two-component sensor histidine kinase	3.3	-1.7	9.39E-05
CDR20291_*3128*	two-component response regulator	3.4	-1.7	0.0365
agrC	sensor histidine kinase virs	20.1	-4.3	2.17E-18
agrA	dna-binding response regulator	14.9	-3.9	4.03E-08
**Riboswitch**				
Cd1	5' untranslated region of flagellar operon (1)	5.3	-2.4	1.22E-13
**Intergenic regions**				
CDR20291_*229*/_*230*		10.3	-3.4	0.0309
CDR20291_*271*/_*272*		4.2	-2.1	0.0007
CDR20291_*439*/_*440*		26.1	-4.7	2.98E-33
CDR20291_*684*/_*685*		3.0	-1.6	0.0003

Supplementary Table 4 List of up-regulated transcripts in R20291 *agrA*76a::CT mutant

**CDS**	**Product**	**Fold change**	**log_2 fold change**	**pvalue-adj**
CDR20291_*2121*	putative regulatory protein	1.8	0.8	0.0477
CDR20291_*2122*	putative regulatory protein	2.6	1.4	0.0056
CDR20291_*3422*	putative ABC transporter, permease protein	6.8	2.8	7.12E-22
CDR20291_*3423*	ABC transporter, ATP-binding protein	3.9	2.0	1.19E-07
CDR20291_*3424*	two-component sensor histidine kinase	7.0	2.8	2.43E-16
CDR20291_*3425*	two-component response regulator	6.7	2.7	1.74E-10
**Intergenic regions**				
CDR20291_*0179/0180*		3.4	1.8	0.0162
CDR20291_*0828/0829*		2.7	1.4	0.0301
CDR20291_*1491/1492*		3.7	1.9	4.06E-07
CDR20291_*2393/2394*		3.4	1.8	0.0342
CDR20291_*3075/c*		2.5	1.3	0.0380

REFERENCES

1. Sudarsan N, Lee ER, Weinberg Z, Moy RH, Kim JN, Link KH, Breaker RR. 2008. Riboswitches in eubacteria sense the second messenger cyclic di-GMP. Science 321:411-413.
